# Perceived Subgroups, TMS, and Team Performance: The Moderating Role of Guanxi Perception

**DOI:** 10.3389/fpsyg.2019.02655

**Published:** 2019-11-29

**Authors:** Mingqiao Luan, Hong Ren, Xuguang Hao

**Affiliations:** ^1^Business School, University of International Business and Economics, Beijing, China; ^2^Sheldon B. Lubar School of Business, University of Wisconsin–Milwaukee, Milwaukee, WI, United States

**Keywords:** perceived subgroups, TMS, guanxi (informal social network), team performance, Chinese context

## Abstract

As teams become increasingly common for organizations to accomplish key objectives, improving team performance is a critical challenge for both practitioners and researchers. As researchers have converged on the notion that team performance is strongly influenced by subgroups, scholars have begun to explore how perception of subgroups influence team performance. Thus, in this study, we examined how perceived subgroups influenced the team transactive memory system (TMS), and hence team performance. We also proposed the moderating role of guanxi perception on the relationship between perceived subgroups and TMS. Utilizing two-wave multi-source data from 87 working teams in a Chinese central government-owned corporation, and based on multiple (moderator) hierarchical regression analyses, our results demonstrated that perceived subgroups were a negative predictor of TMS and team performance, and TMS mediated the negative relationship between perceived subgroups and team performance. That is, perceived subgroups inhibited team performance by blocking the development of a robust TMS. In addition, guanxi perception acted as a positive moderator, mitigating the negative relationship between perceived subgroups and TMS. Furthermore, the moderated mediation analysis of the integrative model revealed that the indirect effect of perceived subgroups on team performance via TMS was contingent on guanxi perception. Overall, our findings identified the pivotal role of perceived subgroups, TMS, and guanxi perception in working teams in the Chinese context.

## Introduction

Teams increasingly constitute the dominant mode of knowledge production in organizations (Wuchty et al., 2007). However, as team members tend to categorize themselves as smaller collectives based on a variety of characteristics, a team may break into subgroups (Cronin et al., 2011), which may hinder their capacity to fully realize their synergistic potential. Consequently, understanding the effects of such subgroup formation and perception on organizational processes and performance has now become critical (Cronin et al., 2011).

Perceived subgroups refer to group members’ recognition of subgroups splits within the group (Shemla et al., 2016). Previous research has supported the expectation that perception of subgroups can exert deleterious effects on team processes as well as team outcomes (Jehn and Bezrukova, 2010; Cronin et al., 2011; Carton and Cummings, 2012; Shen et al., 2016). For instance, Carton and Cummings (2013) examined the “self-reinforcing” effects of perceived subgroups on affective integration, such that perceived subgroups decreased trust, respect, and liking among teammates, which further reinforced subgroup splits. Shen et al. (2016) also argued that perceived subgroups exposed subgroups to frustration, anxiety, and hostility by members of other subgroups. Overall, prior research suggests that perceived subgroups are detrimental to team dynamics and effectiveness (Jehn and Bezrukova, 2010; Cronin et al., 2011).

Besides the direct relationship between perceived subgroups and team outcomes, researchers have also examined different mediators to specify the indirect effects. For instance, Jehn and Bezrukova (2010) focused on the mediating role of coalition and intragroup conflict through which perceived subgroups negatively affect member satisfaction and group performance. Similarly, Pearsall et al. (2008) demonstrated that emotional conflict mediated the relationship between perceived subgroups and team creativity. More recently, scholars have begun to focus on the mediating role of TMS between perceived subgroups and team performance. For instance, Shen et al. (2016) proposed that TMS partially mediated the effect of perceived subgroups on team performance in distributed teams, while Seong et al. (2015) explored that perceptions of group-level fit worked through TMS to influence team performance.

Transactive memory system refers to team members’ division of cognitive labor for learning, storing, and communicating knowledge and expertise required to complete team tasks (Lewis, 2003). Drawing on the literature of knowledge management, organizations can be treated as information processing systems which gather, interpret, and synthesize information to make organizational decisions (Tushman and Nadler, 1978). Teams, as information processing subunits in organizations, need to deal with problem solving and coordination problems in the process of knowledge production (Tushman and Nadler, 1978). Given that the basic function of TMS is to provide a knowledge reservoir for team members to facilitate and distribute information, we propose that TMS can serve as the mediator between perceived subgroups and team performance. In particular, we suggest that perceived subgroups inhibit teams from developing a robust TMS, which eventually lead to expertise underutilization, and performance deterioration. More importantly, going beyond existing research (e.g., Shen et al., 2016), we also examine a potential contingency factor that influences the relationship between perceived subgroups and TMS in the Chinese context.

Existing research has also identified different moderators which may influence subgroups’ effects in teams. According to Joshi and Roh (2009), contextual factors can set specific constraints and opportunities that either enhance or mitigate the direct effects of work team diversity on performance. Contextual factors can be classified into different levels, such as team level, organizational level, and extraorganizational level (Joshi and Roh, 2009). Most previous research on subgroups has focused on team-level contextual factors. For instance, Ren et al. (2015) suggest that different types of network ties across subgroups may activate or deactivate dormant faultlines, thereby leading to opposite team outcomes. Specifically, bridging friendship ties can mitigate the effects of subgroups on team performance, while breaching animosity ties will strengthen the effects of subgroups on team performance. This finding supports the premise that cross-subgroup friendship and extended contact are associated with more positive outgroup attitudes (Turner et al., 2008). Other team-level variables are also explored as moderators of the relationship between subgroups and team outcomes, such as superordinate identity (Homan et al., 2008; Bezrukova et al., 2009; Jehn and Bezrukova, 2010), cognitive integration (Cronin et al., 2011), and task and goal structures (Meyer et al., 2014).

Despite these findings, an important gap in the literature on perceived subgroups is the lack of consideration of extraorganizational contextual factors within a certain society (Joshi and Roh, 2009). Roberson et al. (2017) suggest that diversity research may benefit from an understanding of the role of cultural contexts. Therefore, in this study, we focus on the Chinese context, and highlight “guanxi perception” as an important extraorganizational contextual factor to investigate how it influences team processes and outcomes. Guanxi, described as interpersonal relationships rooted in the Chinese society in which people tend to gain interests through informal interactions (Dunning and Kim, 2007), helps to define and confirm social network structures among people.

This study makes several contributions to the subgroup and guanxi literatures. First, we highlight the role of perceived subgroups in teams rather than the hypothetical split focused on in the faultline literature (Lau and Murnighan, 1998). Specifically, we propose and examine how perceived subgroups influence team TMS, and thus team performance. Second, we identify an important extraorganizational contextual factor, guanxi perception, in the Chinese context, and examine how it interacts with subgroup dynamics. We argue that when perception of subgroups hinders inter-subgroup communication, teams’ guanxi perception will motivate team members to interact across subgroups, diminish communication blocks, and enhance understanding between subgroups. Finally, this study enriches the team literature by investigating how perceived subgroups, guanxi perception, and TMS holistically affect team performance in the Chinese context.

## Hypotheses Development

**FIGURE 1:**
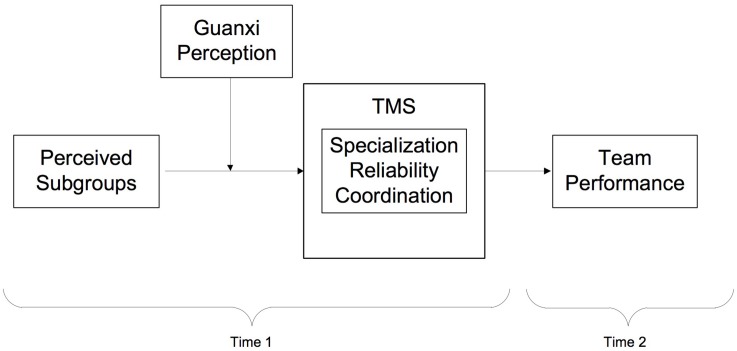
Hypothesized model.

In this section, we propose several possible relationships between perceived subgroups, TMS, guanxi perception, and team performance (see Figure 1).

### Perceived Subgroups in Teams

According to social categorization (Tajfel, 1981) and similarity/attraction theories (Byrne et al., 1971), people with similar attributes within the same group tend to cluster together, create faultlines, and form subgroups (Lau and Murnighan, 1998). Consistent with diversity studies, faultlines or subgroups based on demographics such as age, gender, race, etc. are most widely investigated (Thatcher and Patel, 2012). Scholars have also classified subgroups as identity-based or knowledge-based, suggesting different effects based on the configurational properties of subgroups (Carton and Cummings, 2013; Spoelma and Ellis, 2017).

Our study argues that despite the complex configurational properties of subgroups, once team members perceive subgroups within their teams, these perceptions will negatively influence team dynamics and outcomes. Specifically, perceived subgroups can be defined as to what extent team members recognize the subgroup split within the team (Shemla et al., 2016). The growing body of research on perceived differences demonstrates that people’s reaction is based on perception of reality rather than reality *per se* (Shemla et al., 2016). Therefore, we contend that the demographic composition within a group is not necessarily related to perceived differences. In other words, congruence between potential subgroups and perceived subgroups is not a foregone conclusion (Zellmer-Bruhn et al., 2008). Importantly, the perception of group composition is one of the most significant elements that drives the effects of diversity on group outcomes (Greer and Jehn, 2007). When subgroups are perceived by group members, biases will be more likely raised between subgroups due to members’ in-group and out-group categorization (Chatman et al., 1998). Thus, it is essential to recognize to what extent individuals actually identify the existence of subgroups (Thatcher and Patel, 2012). We argue that team members’ perception of subgroups will negatively influence team performance.

Empirical studies have demonstrated the negative effects that perceived subgroups bring to both individuals and teams. For instance, Shemla and Wegge (2019) found that perceived subgroups were negatively related to information elaboration because perception of subgroup splits led to difficulties in exchanging and integrating information across subgroups. Greer and Jehn (2007) found that perceived subgroups were positively related to conflict between subgroups because subgroup identification triggers ingroup–outgroup biases. In addition, asymmetry of perceived subgroups can cause frustration and withdrawal of group members (Polzer et al., 2002), and further lead to poor decision-making (Greer and Jehn, 2007). Overall, perceived subgroups contribute to an obstruction of communication channels between subgroups, and ultimately lower team performance. Hence, the following hypothesis is proposed:

Hypothesis 1. Perceived subgroups are negatively related to team performance.

### Transactive Memory System (TMS)

Transactive memory system is originally defined as a set of knowledge and information possessed by individuals and then combined into group assets through a process of encoding, storing, retrieving, and communicating (Wegner, 1987). TMS is a three-dimensional construct, including specialization, reliability, and coordination (Wegner, 1987). Specialization refers to the existence of specialized knowledge in teams; reliability refers to team members’ belief regarding whether others’ knowledge is reliable; coordination refers to the team’s ability to smoothly and effectively coordinate knowledge (Wegner, 1987). Research has demonstrated that a well-developed TMS offers many potential benefits for team performance (Lewis, 2004; Zhang et al., 2007; Bachrach et al., 2019). First, TMS helps to increase team performance by allowing team members to quickly become familiar with people, tasks, knowledge, and problems (Liang et al., 1995). Second, when acute stress was proven to have a negative influence on team performance (Ellis, 2006), TMS acted as a cognitive and behavioral mechanism, positively mediating the relationship between physical and psychological stress and team performance. Furthermore, Choi et al. (2010)’s research on knowledge-based teams demonstrated that TMS was positively related to knowledge sharing and application, which in turn facilitated team performance.

Transactive memory system stresses not only to what extent team members perceive others’ expertise (who knows what), but also emphasizes the process of differentiation and distribution of team-specialized knowledge (Huber and Lewis, 2010). TMS can vary in terms of the accuracy of team members’ perceptions about others’ expertise, the degree of a shared representation of the system, and the degree of team members’ participation (Brandon and Hollingshead, 2004). The optimal state of TMS occurs when all of the information coming into a group is fully allocated, stored, and shared by experts in that group (Brandon and Hollingshead, 2004).

Perceived subgroups inhibit team performance by blocking the development of a robust TMS. In a diverse team with perceived subgroups, social categorization amplifies perceived similarities within subgroups, making it hard for teammates to identify and acknowledge specialized knowledge in the whole team (O’Leary and Mortensen, 2010). Besides, subgroup boundaries impede knowledge coordination and reduce perceived credibility between subgroups, and thus hinder the development of TMS (O’Leary and Mortensen, 2010). Rupert et al. (2016) found that teams with distant subgroups were less likely to build an accurate TMS, which in turn impaired team learning. To be more specific, with regard to specialization, perceived subgroups lead team members to develop redundant or overlapping knowledge instead of differentiated expertise (Lewis, 2003), which inhibit the specialized knowledge to be distributed in the whole teams. With regard to reliability, the existence of perceived subgroups makes it hard for team members to judge the credibility and quality of others’ knowledge (Shen, 2009). In other words, teams with perceived subgroups have lower levels of trust (Carton and Cummings, 2013), which causes members to suspect the credibility of knowledge held by other subgroups. Finally, with regard to coordination, perceived subgroups may cause information to be only held within their subgroups, impairing the effectiveness of coordination. Therefore, we argue that perceived subgroups negatively influence team performance by inhibiting teams from developing a robust TMS.

Hypothesis 2. TMS mediates the relationship between perceived subgroups and team performance.

### Guanxi Perception

“Guanxi” refers to the phenomenon that people establish connections to secure favors and interests in interpersonal relationships (Park and Luo, 2001; Dunning and Kim, 2007). Guanxi originated in Confucianism, which defines Chinese social philosophy and hierarchical structures (Park and Luo, 2001). The establishment of guanxi refers to the connection between independent individuals or parties that enables a bilateral flow of personal or social transactions (Lin, 2011).

Theoretically, guanxi is rooted in social capital theory (Park and Luo, 2001). Bourdieu (1986) defined social capital as relationships of mutual acquaintance recognition aggregated by durable network resources. In the framework of social capital theory, a set of nodes (e.g., persons, groups) are linked by a set of social relationships (e.g., overlapping memberships). Through guanxi’s three notions categorized by Li (2007) (which are weak, strong, and total), guanxi can be considered as the “Chinese version of social capital” rooted in the unique institutional context of China. Compared with Western networks, which entail the exchange of equivalent value, Chinese guanxi often links people across uneven ranks, with the lower party requesting favors from higher parties. Moreover, guanxi stresses the importance of face (mianzi) and reciprocity (renqing), whereas Western networks are based on emotional attachments (Park and Luo, 2001). Consistent with the social capital perspective, resource dependence theory (Salancik and Pfeffer, 1978) can be utilized to explain the activation of guanxi. From that perspective, one cannot generate all needed resources on his or her own; as a result, interpersonal efforts must be exerted to exchange and obtain resources. Since diverse groups provide more resources for people to exchange, guanxi may affect the flow of resources in groups, and finally affect group functioning (Park and Luo, 2001).

This study focuses on guanxi in group settings, and explores the effects of team members’ perception of guanxi on group processes and outcomes. We define guanxi perception as team members’ perception about the importance of guanxi networks. Chen and Tjosvold (2007)’s research highlighted the positive value of Chinese guanxi by demonstrating that personal guanxi promotes employees’ constructive controversy with their foreign managers and benefits working groups by providing an open-minded discussion perception. In addition, Chen and Agrawal (2018) suggested that certain level of guanxi between diverse team members smoothed the process of coordination, which engaged team members to learn together as a team. Thus, this study proposes that guanxi perception would play an important role in teams with perceived subgroups in the Chinese context. On the one hand, based on in-group favoritism, Chinese people have a greater tendency to categorize insiders and outsiders (Farh et al., 1998). On the other hand, based on social capital theory, Chinese people possess a strong tendency to establish networks with outsiders. These two contradictory tendencies may result in complex group outcomes. Perceived subgroups may disintegrate teams and cause between subgroup conflicts, but guanxi perception may link subgroups by enhancing coordination and mutual trust. In other words, teams with higher perception of guanxi are more likely to utilize guanxi to help establish connections between subgroups for the purpose of promoting mobility of social interactions (Yeung and Tung, 1996). Through the development of guanxi, subgroups are able to acquire resources and valuable information (Moses, 2007), increase interdependence and collaboration (Park and Luo, 2001), augment the speed of problem-solving (Kotabe et al., 2003), and improve the quality of decision-making (Peng and Luo, 2000).

Furthermore, as previously discussed, perceived subgroups have a negative relationship with TMS. Guanxi perception may mitigate this negative effect. First, guanxi enables team members to maintain a relatively close relationship and, as a result, this closeness makes people know many things about each other’s knowledge and expertise (Wegner et al., 1991). According to Wegner et al. (1991), each partner can enjoy the benefits of the group’s memory by assuming responsibility for remembering just certain categories of knowledge. Such knowledge can be retrieved from the partner when needed. As a result, team members can concentrate on the development of their own expertise, and leave those unfamiliar domains to other team members who are specialized in. Second, TMS requires effective communication and coordination among team members as a premise (Lewis, 2004), and guanxi perception drives team members to establish connections across subgroups. Huang et al. (2013)’s study investigated the role of social ties on TMS, and concluded that expressive social ties exacerbate the influence of the coordination dimension of TMS on knowledge quality. Likewise, as group members value guanxi, there will be more information flowing across subgroups, facilitating the establishment of effective communication and knowledge transformation (Huber and Lewis, 2010). Third, TMS is built on the premise of mutual trust among team members because credibility of expertise is based on credibility of people. While perceived subgroups hinder mutual trust between in-groups and out-groups, guanxi perception enhances reliability and reduces losses of TMS.

On the other hand, when guanxi perception is low, the negative effect of perceived subgroups on TMS will be more pronounced. First, due to self-categorization (Tajfel, 1981), perceived subgroups reduce team members’ motivation to establish effective TMS. In addition, the motivation will further decrease when team members do not value guanxi. That is, when guanxi perception is low, team members will have low motivation to interact across subgroups in order to better exchange resources across subgroups, which in turn inhibits the team from developing TMS. Second, as mentioned above, perceived subgroups reduce reliability of certain specialized knowledge owned by subgroups, and the situation will get worse if team members do not value guanxi. Since guanxi is established through long-term trust (Luo, 2007), when guanxi perception is low, members will have even lower motivation to trust the others’ knowledge.

Therefore, we propose that guanxi perception will mitigate the negative effects of perceived subgroups on TMS. Under the circumstance that people have the perception of subgroup splits, guanxi perception plays a key role in influencing team members’ way of interaction. Specifically, guanxi perception can serve as a bridging channel, cutting across subgroups, mitigating subgroups’ effects, and improving the establishment of TMS (Inkpen and Tsang, 2005). Hence, the following hypothesis is proposed:

Hypothesis 3. Guanxi perception mitigates the negative relationship between perceived subgroups and TMS.

## Materials and Methods

### Data Collection

Our study was approved by the University of International Business and Economics Ethics Committee. To test our hypotheses, we surveyed employees in management and production teams of a Chinese central government-owned corporation, who had 21 subsidiaries throughout the country. Its main business ranged from electricity, railway, harbor, shipping, coal mining, to coal chemistry. It ranked in the top 200 in the 2015s Forbes. In order to distribute our questionnaires, we contacted several leaders in that corporation to support our research, including the vice president of the corporation at its headquarters, and six members of the board of directors of its subsidiaries. After obtaining their permission, we went to six of their subsidiaries’ headquarters to distribute our questionnaires to their management teams and production teams. The first wave of surveys was collected in April 2017, and the second wave of data was collected 4 months later.

Managers of the human resource departments coordinated the allocation processes. Hyperlinks to web surveys were sent to their “WeChat” (the most popular online chatting service in China) working groups, as well as their internal office assistant systems. We stayed in their working places throughout the allocation and collection processes for 2 weeks in the first wave, monitoring the response rate and also providing instructions to participants and answers to any of their questions. The survey contained questions related to perceived subgroups, TMS, guanxi perception, and some demographics.

Since we administered our questionnaires in the Chinese context, we translated the scales of perceived subgroups and guanxi perception into the Chinese language. The process of translation followed the four steps of scrupulous translation, which were forward translation, assessment, backward translation, and reassessment (Song et al., 2009). For items of TMS, we applied Zhang et al. (2007)’s translation, which were mature and tested in practice. Four months later, we collected the six subsidiaries’ performance appraisal archival data as a measure of team performance.

### Sample

We obtained access to 1,252 team members to complete our surveys and matched those team members into 134 teams with the assistance of six human resources staff members. Removing groups whose size was smaller than three and groups whose within-team response rates were lower than 80%, we obtained 102 groups with 589 surveys, representing an effective response rate of 47% at the group level. Among the respondents, 53% were males. Ages ranged from 24 to 59 (*M* = 37, *SD* = 7.3), and tenure ranged from 1 to 41 years (*M* = 14, *SD* = 8.6). In terms of education level, 3.2% had a high school degree, 14.8% had a diploma, 71.8% had a bachelor’s degree, and 10.2% had a master’s degree. Team sizes ranged from 4 to 18. Seventy-seven teams were in management, whereas 25 teams were in production. Four months later, we contacted the HR department of each subsidiary to obtain the team performance measure and scoring standards. Complete data were collected for 87 teams, representing a response rate of 85%.

### Measures

#### Perceived Subgroups

Perceived subgroups were measured based on three items adopted from Earley and Mosakowski (2000)’s subgroup perceptions measure and Jehn and Bezrukova (2010)’s activated faultlines measure. Earley and Mosakowski (2000)’s measure reflects team members’ perceptions of subgroup formation. On the other hand, although named activated faultlines, Jehn and Bezrukova (2010)’s measure also assesses to what extent “group members perceived a division of the group into separate subgroups” (Jehn and Bezrukova, 2010, p. 30). Therefore, building on these two studies, we used the following three items to measure perceived subgroups: “My team split into subgroups during daily work,” “My team divided into subsets of people during daily work,” and “My team broke into two groups during daily work.” Responses were given on a seven-point Likert-type scale, ranging from 1 = very inaccurate to 7 = very accurate. Omega total was 0.91 (see Table 1). We calculated the intraclass correlation coefficient (James, 1982) to justify aggregation. The ICC values were: 0.16 (ICC1), 0.53 (ICC2). Thus, individual responses were aggregated to a team level score for analysis.

**TABLE 1:** Descriptive statistics and inter-correlations.

**Variables**		***M***	***SD***	**1**	**2**	**3**	**4**	**5**	**6**	**7**	**8**	**9**	**10**	**11**	**12**	**13**	**14**
1	Perceived subgroups (T1) ^b^	2.50	1.07	0.91^a^													
2	TMS (T1)	5.95	0.62	–0.31^∗∗^	0.96												
3	TMS specialization	5.84	0.71	−0.24^∗^	0.96^∗∗∗^	0.90											
4	TMS reliability	6.05	0.62	–0.30^∗∗^	0.95^∗∗∗^	0.86^∗∗∗^	0.91										
5	TMS coordination	6.00	0.62	–0.37^∗∗∗^	0.92^∗∗∗^	0.81^∗∗∗^	0.84^∗∗∗^	0.86									
6	Guanxi perception (T1)	3.50	0.88	0.49^∗∗∗^	–0.12	–0.06	–0.13	−0.19^∗^	0.84								
7	Team performance (T2) ^c^	–0.08	1.13	–0.31^∗∗^	0.29^∗∗^	0.28^∗∗^	0.27^∗^	0.27^∗^	–0.18	–							
8	Fls (age and gender)	0.09	0.08	0.14	–0.06	–0.06	–0.04	–0.08	0.03	–0.05	–						
9	Fls (tenure and education)	0.08	0.08	–0.01	0.09	0.11	0.09	0.05	–0.07	–0.07	0.37^∗∗∗^	–					
10	Age diversity	5.61	2.19	0.10	–0.13	–0.15	–0.13	–0.10	0.07	0.10	0.18	–0.05	–				
11	Gender diversity	0.27	0.21	–0.06	0.02	0.01	0.06	0.01	–0.00	0.10	–0.12	0.11	–0.17	–			
12	Tenure diversity	7.45	3.34	–0.03	0.06	0.03	0.08	0.08	0.04	0.06	0.15	0.01	0.76^∗∗∗^	–0.07	–		
13	Education diversity	0.34	0.20	–0.09	0.00	–0.01	0.00	0.02	–0.04	–0.12	–0.04	–0.11	0.11	0.17	0.22^∗^	–	
14	Team size	5.81	3.03	–0.05	0.14	0.14	0.13	0.13	–0.10	−0.22^∗^	–0.09	0.01	0.08	0.18	–0.01	0.14	–

*^*a*^Reliabilities (Omega total) are indicated along the diagonal; ^*b*^T1 = time 1; ^*c*^T2 = time 2; ^∗^*p* < 0.05; ^∗∗^*p* < 0.01; ^∗∗∗^*p* < 0.001.*

#### Transactive Memory System

We measured TMS by using the 15-item scale developed by Lewis (2003), containing three dimensions: specialization, reliability, and coordination (Wegner, 1987). The response format ranged from 1 = strongly disagree to 7 = strongly agree. Examples of items were “Each team member has specialized knowledge of some aspect of work” (specialization), “I trusted that other members’ knowledge was credible” (reliability), and “Our team worked together in a well-coordinated fashion” (coordination). Based on a factor analysis, we removed five items with low loadings. Omega total of the three subscales were 0.90, 0.91, and 0.86, respectively. Omega total for the overall TMS was 0.96 (see Table 1). ICC values were 0.22 (ICC1), 0.61 (ICC2) for TMS; 0.15 (ICC1), 0.49 (ICC2) for TMS-specialization; 0.14 (ICC1), 0.50 (ICC2) for TMS-reliability; 0.23 (ICC1), 0.62 (ICC2) for TMS-coordination, justifying aggregation.

#### Guanxi Perception

Guanxi perception was measured by scales developed by Dunning and Kim (2007). After deleting items whose loadings were low in factor analyses, six items were retained to reflect our measure. Items used in our survey were “My network of contacts does not consist of only who I know but also includes those that my contacts know.”, “I can make use of my contacts’ contacts as long as I have a good relationship with my contacts.”, “People should help one another at all times; you never know when you might need their help.”, “A personal connection is developed and reinforced through personal care and commitment.”, “It is fair that people can gain favors/benefits by depending on their network of contacts.”, and “It is natural that I give favors to and receive favors from my network of contacts.” The response format ranged from 1 = disagree strongly to 7 = strong agree. Omega total was 0.84 (see Table 1). We aggregated individuals’ guanxi perceptions to the team level to indicate each team’s guanxi perception.

#### Team Performance

Team performance was rated by each team’s human resource and enterprise management department. Among the three subsidiaries of 87 teams, the scoring rules were similar. Team performance scores were a weighted average of peer-to-peer scoring, leader scoring, and staff members’ daily performance scores (such as teams’ average attendance and violation of rules and regulations). We standardized the scores by subtracting each subsidiary’s mean score, and dividing the new results by each of the standard deviations.

#### Control Variables

Faultline strength represents the salience of boundaries between potential subgroups (Carton and Cummings, 2013). Consistent with previous work (Bezrukova et al., 2009), we controlled for identity-based faultline strength (members’ alignment on age and gender) and knowledge-based faultline strength (members’ alignment on education level and tenure). We calculated the two faultlines following the procedure described by Shaw (2004) and based on the SAS program developed by Chung et al. (2006).

We also controlled for heterogeneity in age, gender, education level and tenure because teams with the same configurational properties can have different faultline strength (Lau and Murnighan, 1998). We used Blau (1977)’s heterogeneity index to measure diversity for categorical variables such as gender and education level. We used the coefficient of variation to measure diversity for continuous variables such as age and tenure. In addition, we controlled for team size because the larger a team is, the more likely it is to split into subgroups (Shaw, 2004). Team size was acquired by subsidiaries’ archival materials. Tenure was the average length of time that team members had worked in that organization. Education level was coded as: 1 = high school, 2 = diploma, 3 = bachelor, 4 = master, 5 = Ph.D.

## Results

### Confirmatory Factor Analyses

We first conducted a confirmatory factor analysis (CFA) to demonstrate discriminant validity among our three theoretical variables measured with multiple scale items by team members: perceived subgroups, guanxi perception, and TMS. Perceived subgroups and guanxi perception were loaded in one factor, respectively, whereas TMS was loaded on three factors. The results suggested that our hypothesized model with five correlated latent factors has a good fit [χ^2^ = 351.82, df = 224, *p* < 0.001,RMSEA = 0.075, 90% confidence interval of RMSEA = (0.059, 0.069), GFI = 0.83, AGFI = 0.79, CFI = 0.96, IFI = 0.96, NNFI = 0.95, and TLI = 0.98], and fit the data better than the alternative nested models.

### Hypothesis Testing

Descriptive statistics were presented in Table 1. Multiple regression analyses were used and reported in Tables 2, 3.

**TABLE 2:** Result of regression analysis for team performance (T2).

**Predictors**	**Standardized regression coefficients**						
	**Team performance (T2)**						
	**Step 1**	**Step 2**	**Step 3**	**Step 4**	**Step 5**	**Step 6**	**Step 7**
**Controls**							
Fls (age and gender)	–0.71	0.11	0.12	0.48	0.47	0.30	0.50
Fls (tenure and education)	–01.00	–1.37	–1.40	–1.90	–2.07	–1.71	–1.67
Age diversity	0.01	0.05	0.05	0.11	0.12	0.11	0.09
Gender diversity	0.94	0.95	0.96	1.08^∗^	1.12^∗^	1.01	1.05
Tenure diversity	0.04	0.01	0.01	–0.03	–0.03	–0.03	–0.02
Education diversity	–0.88	–0.85	–0.83	–0.61	–0.62	–0.64	–0.66
Team size	−0.08^∗^	−0.10^∗^	−0.10^∗^	–0.12^∗∗^	–0.12^∗∗^	–0.12^∗∗^	–0.11^∗∗^
Perceived subgroups (T1)		–0.34^∗∗^	−0.31^∗^	–0.22	–0.23	–0.23	–0.23
Guanxi perception (T1)			–0.06	–0.10	–0.12	–0.09	–0.07
TMS (T1)				0.52^∗∗^			
TMS specialization					0.48^∗∗^		
TMS reliability						0.46^∗^	
TMS coordination							0.41^∗^
Overall R^2^	0.10	0.21	0.21	0.28	0.29	0.27	0.25
Adjusted R^2^	0.02	0.12	0.12	0.19	0.20	0.17	0.15
Δ R^2^		0.10	0.00	0.07	0.08	0.05	0.03
Overall F	1.27	2.57^∗^	2.27^∗^	2.98^∗∗^	3.17^∗∗^	2.76^∗∗^	2.56^∗^
df	79	78	77	76	76	76	76

*^∗^*p* < 0.05; ^∗∗^*p* < 0.01.*

**TABLE 3:** Result of regression analysis for TMS.

**Predictors**	**Standardized regression coefficients**															
	**TMS**				**TMS specialization**				**TMS reliability**				**TMS coordination**			
	**Step 1**	**Step 2**	**Step 3**	**Step 4**	**Step 5**	**Step 6**	**Step 7**	**Step 8**	**Step 9**	**Step 10**	**Step 11**	**Step 12**	**Step 13**	**Step 14**	**Step 15**	**Step 16**
**Controls**																
Fls (age and gender)	–0.59	–0.31	–0.27	–0.11	–0.70	–0.47	–0.41	–0.24	–0.36	–0.08	–0.05	–0.07	–0.68	–0.31	–0.29	–0.14
Fls (tenure and education)	0.75	0.64	0.68	0.71	1.07	0.99	1.06	1.09	0.59	0.49	0.52	0.55	0.47	0.33	0.35	0.38
Age diversity	–0.14^∗∗^	–0.12^∗∗^	–0.12^∗∗^	−0.09^∗^	–0.15	–0.13^∗∗^	−0.13^∗^	−0.10^∗^	–0.14^∗∗^	–0.12^∗∗^	–0.12^∗∗^	−0.10^∗^	–0.12^∗∗^	−0.10^∗^	−0.09^∗^	–0.07
Gender diversity	–0.21	–0.21	–0.22	–0.27	–0.30	–0.30	–0.32	–0.37	–0.06	–0.06	–0.07	–0.11	–0.23	–0.23	–0.23	–0.28
Tenure diversity	0.08^∗∗^	0.07^∗^	0.07^∗^	0.05	0.09^∗^	0.08^∗^	0.07^∗^	0.05	0.09^∗∗^	0.08^∗∗^	0.08^∗∗^	0.06^∗^	0.08^∗∗^	0.06^∗^	0.06^∗^	0.04
Education diversity	–0.17	–0.21	–0.21	–0.10	–0.20	–0.23	–0.23	–0.10	–0.20	–0.24	–0.24	–0.15	–0.11	–0.16	–0.16	–0.04
Team size	0.04^∗^	0.03^∗^	0.04^∗^	0.04	0.04^∗^	0.05	0.05^∗^	0.04	0.04	0.03	0.04	0.03	0.04	0.03	0.03	0.03
Perceived subgroups (T1)		–0.15^∗∗^	−0.18^∗^	–0.76^∗∗∗^		−0.12^∗^	−0.18^∗^	–0.83^∗∗^		−0.14^∗^	−0.17^∗^	–0.62^∗∗^		–0.19^∗∗∗^	–0.21^∗∗^	–0.79^∗∗∗^
Guanxi perception (T1)			0.07	–0.28			0.11	–0.29			0.06	–0.22			0.04	−0.33^∗^
PS^∗^GC ^a^				0.15^∗∗^				0.17^∗∗^				0.11^∗∗^				0.15^∗∗^
Overall R^2^	0.13	0.19	0.20	0.27	0.13	0.16	0.17	0.24	0.13	0.19	0.19	0.23	0.10	0.20	0.21	0.27
Adjusted R^2^	0.07	0.12	0.12	0.19	0.06	0.09	0.09	0.15	0.07	0.12	0.12	0.15	0.03	0.14	0.14	0.20
Δ R^2^		0.05	0.00	0.07		0.03	0.00	0.06		0.05	0.00	0.03		0.11	0.00	0.06
Overall F	2.07^∗^	2.8^∗∗^	2.57^∗^	3.31^∗∗^	1.96	2.18^∗^	2.09^∗^	2.81^∗∗^	2.03	2.67^∗^	2.41^∗^	2.75^∗∗^	1.58	3.00^∗∗^	2.66^∗∗^	3.45^∗∗∗^
df	94	93	92	91	94	93	92	91	94	93	92	91	94	93	92	91

*^∗^*p* < 0.05; ^∗∗^*p* < 0.01; ^∗∗∗^*p* < 0.001.*

#### Effects of Perceived Subgroups in Working Teams

According to Hypothesis 1, perceived subgroups will be negatively related to team performance. To test this hypothesis, we regressed the seven control variables and perceived subgroups on team performance. As shown in Table 2, Step 2, perceived subgroups were negatively related to team performance (β = −0.34, *p* < 0.01). Thus, Hypothesis 1 was supported.

#### Effects of TMS in Working Teams

We proposed in Hypothesis 2 that TMS would mediate the relationship between guanxi perception and team performance. We regressed team performance on TMS together with the control variables and perceived subgroups. Evidence showed that (Table 2, step 4) TMS was positively related to team performance (β = 0.52, *p* < 0.01) and perceived subgroups became non-significant (β = −0.22, *p* > 0.05). In addition, among the control variables included in our model for team performance, team size was negatively related to team performance (β = −0.12, *p* < 0.05). On the other hand, we also regressed TMS on perceived subgroups together with the control variables. As shown in Table 3, Step 2, step 6, step 10, and step 14, perceived subgroups were negatively related to TMS (β = −0.15, *p* < 0.01), TMS specialization (β = −0.12, *p* < 0.05), TMS reliability (β = −0.14, *p* < 0.05), and TMS coordination (β = −0.19, *p* < 0.001). Thus, Hypothesis 2 was supported.

#### Effects of Guanxi Perception in Working Teams

**FIGURE 2:**
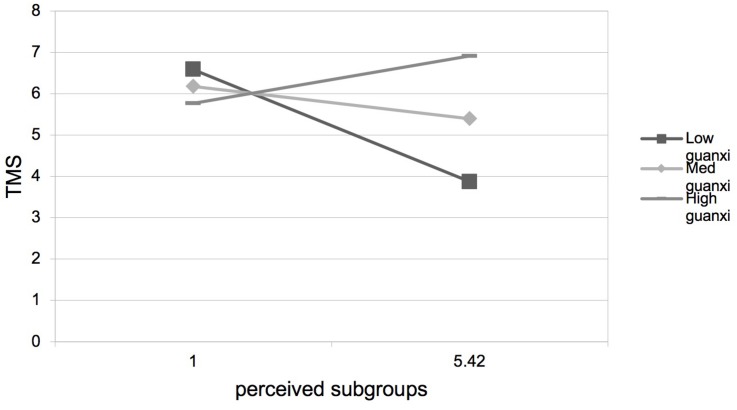
Interaction between perceived subgroups and guanxi perception on TMS.

**FIGURE 3:**
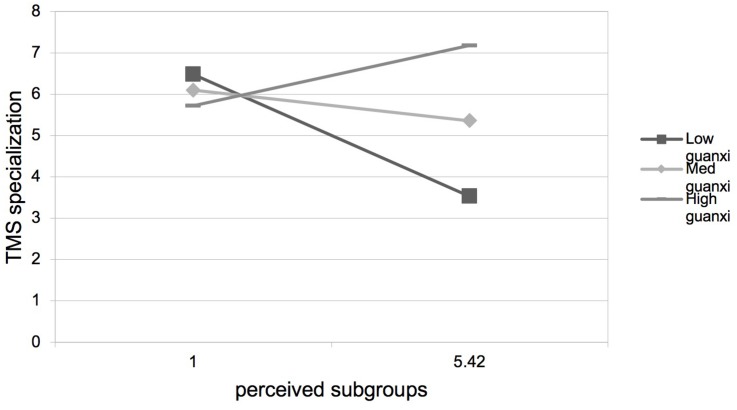
Interaction between perceived subgroups and guanxi perception on TMS specialization.

**FIGURE 4:**
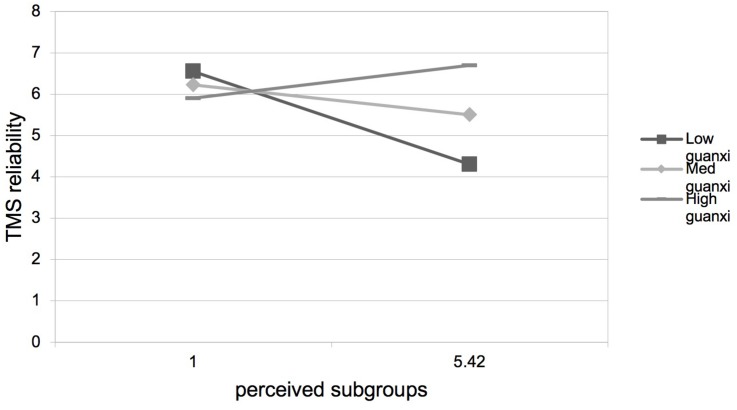
Interaction between perceived subgroups and guanxi perception on TMS reliability.

**FIGURE 5:**
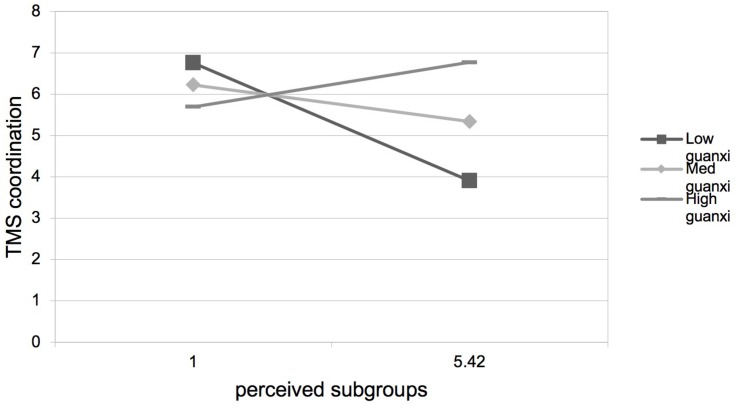
Interaction between perceived subgroups and guanxi perception on TMS coordination.

Hypothesis 3 predicted that guanxi perception would positively moderate the negative relationship between perceived subgroups and TMS. As presented in Table 3, step 4, step 8, step 12, and step 16, the interactions between perceived subgroups and guanxi perception were positive and significant on TMS (β = 0.15, *p* < 0.01), TMS specialization (β = 0.17, *p* < 0.01), TMS reliability (β = 0.11, *p* < 0.01), and TMS coordination (β = 0.15, *p* < 0.01). We plotted the interaction at two levels of guanxi perception. As shown in Figures 2–5, the negative relationships between perceived subgroups and the three dimensions of TMS were more pronounced when guanxi perception was low. Actually, the relationship between perceived subgroups and the three dimensions of TMS became positive when guanxi perception was high. We further conducted simple slopes tests (Aiken et al., 1991). Our results confirmed that guanxi perception acted as a positive moderator. When guanxi perception was low, perceived subgroups were more negatively related to TMS (β = −0.06, *p* < 0.01), TMS specialization (β = −0.08, *p* < 0.01), TMS reliability (β = −0.06, *p* < 0.01), and TMS coordination (β = −0.06, *p* < 0.01). On the other hand, when guanxi perception was high, perceived subgroups were positively related to TMS (β = 0.19, *p* < 0.01), TMS specialization (β = 0.21, *p* < 0.01), TMS reliability (β = 0.19, *p* < 0.01), and TMS coordination (β = 0.18, *p* < 0.01). In other words, Hypothesis 3 was supported.

#### Moderated Mediation Analysis of the Integrative Model

We also investigated whether the indirect effects of perceived subgroups on team performance via TMS were contingent on guanxi perception (see Table 4). Moderated mediation analysis supported this first stage interactive effect (Preacher et al., 2007). Analyses were performed using PROCESS version 2.16.3 for SAS (Hayes, 2017). As shown in Table 4, examination of the conditional indirect effects suggested that perceived subgroups had significant and negative indirect effects on team performance via TMS at low (−1 SD), medium, and high (+1 SD) levels of guanxi perception; the 95% CI’s did not include zero.

**TABLE 4:** Moderated-mediation estimates.

	**Moderated-mediation model**															
	**TMS**				**TMS specialization**				**TMS reliability**				**TMS coordination**			
	**Effect**	**Boot SE**	**Boot LLCI**	**Boot ULCI**	**Effect**	**Boot SE**	**Boot LLCI**	**Boot ULCI**	**Effect**	**Boot SE**	**Boot LLCI**	**Boot ULCI**	**Effect**	**Boot SE**	**Boot LLCI**	**Boot ULCI**
**Conditional indirect effects**																
Low guanxi perception	–0.17	0.09	–0.40	–0.04	–0.16	0.08	–0.38	–0.04	–0.14	0.08	–0.35	–0.02	–0.15	0.09	–0.36	–0.01
Mean guanxi perception	–0.11	0.06	–0.26	–0.02	–0.10	0.06	–0.25	–0.02	–0.10	0.05	–0.23	–0.01	–0.10	0.06	–0.24	–0.01
High guanxi perception	–0.05	0.04	–0.15	0.01	–0.04	0.04	–0.14	0.02	–0.04	0.03	–0.13	0.01	–0.05	0.04	–0.15	0.01
**Index of moderated mediation**																
Guanxi perception	0.07	0.04	0.01	0.16	0.06	0.03	0.01	0.15	0.05	0.03	0.01	0.14	0.06	0.03	0.01	0.14

Overall, the integrative model was supported in our research. That is, perceived subgroups were negatively related to team performance, and the indirect effects of perceived subgroups on team performance via TMS were contingent on guanxi perception.

## Discussion

In this study, we applied a subgroup perspective to understand the structure and functioning of working teams in a Chinese central government-owned corporation. Specifically, we investigated the effects of perceived subgroups and guanxi perception on TMS and team performance. Our findings suggested that perceived subgroups hampered TMS and team performance. However, guanxi perception acted as a moderator, mitigating the negative effect of perceived subgroups on TMS, and thereby team performance. To avoid common method variance, we conducted a two-wave study with multi-source data, which provided less biased evidence of the proposed relationships.

First, faultlines and subgroups offer one approach to examining the effect of team composition. The expectation that faultlines and subgroups based on a variety of demographic factors would negatively influence team dynamics is based on social categorization theory and similarity attraction theory (Byrne et al., 1971; Tajfel, 1981). That is, scholars have relied on homophily on demographic characteristics as a proxy for subgroups and suggest that these demographic subgroups may hinder team performance. However, people’s perception may not reflect demographic homophily (Reagans et al., 2004; Ren et al., 2015). In this study, we argue that what matters is to what extent team members perceive subgroups. In particular, our results demonstrated that perceived subgroups were negatively related to TMS and team performance. This finding also parallels Jehn and Bezrukova (2010)’s assertion that activated (perceived) faultlines tended to generate cooperation obstacles. Therefore, in order to promote team performance in a diverse team, perceived subgroups need to be minimized.

Second, as TMS is regarded as intangible capital possessed by teams, resulting in team effectiveness and efficiency (Zhang et al., 2007), this study highlighted TMS as an important underlying mechanism that mediated the relationship between perceived subgroups and team performance. In a team with perceived subgroups, the self-categorization caused by perception of subgroups hinders information sharing across subgroups, making it difficult for team members to know, trust, and utilize the entire team’s full range of expertise. This offers one theoretical explanation on why perceived subgroups may exert an influence on team performance.

Third, the unique interpersonal relationships in the Chinese society require us to embed our study in its special context. That is, context is important in group settings because it provides the purpose, resources, social cues, norms, and meanings that shape behaviors (Jackson et al., 2003). Our research considers a larger social system (guanxi), within which teams are embedded. Our findings support that the influences of subgroups depend on the degree of informal integration present in teams (Insko et al., 1993). Guanxi perception focuses on team members’ sensitivity to establishing interpersonal relationships across subgroups. It is interesting to note that guanxi perception itself had neither a direct effect on TMS nor team performance evaluated by the managerial departments. This reveals that the concept of “who you know is more important than what you know” (Yeung and Tung, 1996) is insufficient when team members attempt to solve problems in diverse teams. However, the interaction effect between perceived subgroups and guanxi perception affected TMS significantly. That is, in a team with perceived subgroups, what matters is whether team members value guanxi and interpersonal relationships. If they do, they will be able to overcome the obstacles presented by subgroups. Indeed, our results suggest that when teams value guanxi, they can even benefit from the variety of insights presented by different subgroups. This result also parallels the finding by Ren et al. (2015) that friendship bonding ties can help enhance faultlines’ positive effect on team performance. Going beyond the Chinese context, other East Asian countries (e.g., Japan and Korea) which have also been influenced by Confucian philosophical principles are expected to have similar characteristics in social relationships (Yum, 1988). Therefore, the conclusion of our study may also expand to other East Asian countries which share similar relationship beliefs with China.

Overall, our research suggests that the mechanisms of subgroups on team outcomes are more complex than assumed in prior studies that only focused on the direct effect. We highlighted the importance of a potential mediator (e.g., TMS) and a moderator (e.g., guanxi perception) for the indirect effects of perceived subgroups on team performance. Our results shed light on the importance of taking cognitive integration, networks of interpersonal relations and TMS into account while investigating team compositions and their outcomes.

### Limitations and Future Research

Our findings should be interpreted in light of several limitations, which also offer directions for future research. First, this study focused on the influence of perceived subgroups in teams. However, subgroups can be classified into different types. For instance, Carton and Cummings (2012) outlines three underlying factors that characterize subgroups, which are identity (e.g., values), resources (e.g., power, status), and knowledge (e.g., expertise, experience), respectively. In our research, we define perceived subgroups as team members’ general perception of subgroup splits without considering subgroup types. While the global assessment of perceived subgroups allows us to capture the general valence of the perception, more specific measures with regard to what type of subgroups the perception is based on will provide a more nuanced assessment of the influence of perceived subgroups. For instance, previous research has demonstrated that subgroups can be triggered by surface-level attributes, such as age, gender, tenure, specialization, etc. (Lau and Murnighan, 1998), and also by deep level attributes, such as attitudes, values, personalities, or beliefs (Harrison et al., 2002). In this study, we did control for the effects of demographic faultlines and diversity. Still, further study is encouraged to address the basis of subgroups and explore the potential antecedences of subgroup formation.

Relatedly, since we treated the status of team members alike, future study may consider members’ status as possible interventions. For instance, leadership may act as a mechanism that facilitates collective social identities, and may mitigate the negative effects of subgroups on team processes and performance (Kunze and Bruch, 2010). Future research is encouraged to investigate how separation, variety, and disparity (Harrison and Klein, 2007) in subgroups may directly and/or interactively influence team processes and outcomes.

In addition, complete surveys were not obtained for all team members. Teams were included in the analysis if they had more than 80% within-team response rate. We also performed analyses on teams with 100% responses (*n* = 85 for the cross-sectional analysis, *n* = 69 for the longitudinal analysis), and the results were in the same direction as those for 80% responses. However, it reduced our sample size and power to explore the moderating mediation effects.

Furthermore, we investigated guanxi perception as one possible contextual factor that can mitigate the negative effect of perceived subgroups on TMS. Future research may examine other moderators that can facilitate the development of TMS. For instance, Jehn and Bezrukova (2010) proposed that when group members were both committed to and held a superordinate team identity as a primary team identification, the entire group would become more internally cohesive and function more effectively. Future research can specify other team, organizational, and extraorganizational factors to explain effects of team dynamics (Joshi and Roh, 2009). For example, the economic crisis is an important stressor that has a negative impact on employees’ mental health (Mucci et al., 2016). Thus, in the context of an uncertain international situation, researchers can investigate team members’ fear of global crisis (Giorgi et al., 2015) as a potential moderator and explore how external stress might interact with within team subgroups to influence team outcomes.

### Practical Implications

In addition to the theoretical contribution of our results, our study has several practical implications for organizations and managers. First, as perceived subgroups can be detrimental to the development of TMS, it is important for team leaders to highlight the interdependence nature of team work, and encourage team members to communicate with each other across subgroups. This could take the form of social gatherings and activities that allow team members to informally interact with each other. Such relationship building practices may help deal with potential conflicts and misunderstandings among subgroups, and also enable employees to better understand their teammates’ knowledge and facilitate the development of TMS.

Second, team leaders should not be threatened by perceptions of subgroups. Our finding suggests that even if there are perceived subgroups, if team members value the importance of guanxi, the subgroups are able to be integrated into a fully functioning team. Therefore, organizations should promote an organizational culture and climate that value the importance of guanxi. In addition, although guanxi is embodied in the Chinese cultural context, our study can offer important implications for teams in Western cultures. Specifically, the construct of guanxi perception can be expanded to general relationship or network beliefs, or social capital perceptions. That is, team members may learn to use personal attachment to establish norms of group inclusion and increase the commitment of the whole team to a cooperative relationship (Park and Luo, 2001). To encourage team members to develop networks across subgroups, managers may give rewards such as bonus and promotion for utilizing team members’ personal network for team purposes. Besides, managers may hire employees who have extensive networks among the whole team.

Third, our study provided an implication for cross-cultural teams that an understanding of the Chinese culture can help foreign members interact more effectively with Chinese members. With globalization, people from different cultures inevitably work together. Therefore, understanding different cultures can enhance conversation and knowledge transfer in cross-cultural teams. Previous research has argued that cultural differences make understanding and cooperation among team members more challenging (Bond, 2003). However, understanding different cultures can enhance conversation and knowledge transfer in cross-cultural teams. For instance, Chen and Tjosvold (2007) argue that American managers’ understanding of Chinese guanxi is expected to promote open-minded discussion with local employees in international joint ventures.

Finally, management should pay more attention to the importance of TMS. A well-developed TMS can reduce information overlaps and help the whole team to complete complex tasks. It also assists to diagnose inefficiency and team failure. In order to establish an effective TMS, managers may consider and incorporate the implications of team members’ knowledge before building teams. In more details, selecting members who possess complementary knowledge can greatly accelerate the formation of TMS and enable identification and implementation of effective solutions to subgroup formation.

## Conclusion

Research on subgroups has yielded inconsistent findings in recent decades, which necessities the utilization of multiple methods to elucidate the mechanism between the salience of subgroups and work outcomes. In this paper, we review team composition literature with a perceived subgroup perspective. We emphasize the importance of taking guanxi perception into consideration while studying team composition and its effects embedded in a Chinese context.

As the influence of team dynamics continues to attract research focus, it is critical to discover how team members react to their perception of reality rather than reality *per se* (Shemla et al., 2016). This study applies guanxi context to subgroup research as the first step in this effort. In the near future, we hope to see more studies on additional ways that guanxi, as well as other contextual factors, can provide significant value to working teams in organizations.

## Data Availability Statement

The datasets generated for this study are available, upon reasonable request, to any qualified researcher. Requests to access the datasets should be directed to the corresponding author.

## Ethics Statement

This study was carried out in accordance with the recommendations of the University of International Business and Economics Ethics Committee. All subjects gave informed consent prior to participation in the study. All subjects could voluntarily withdraw from participation at any time. The protocol was approved by the University of International Business and Economics Ethics Committee.

## Author Contributions

All authors listed have made a substantial, direct and intellectual contribution to the work, and approved it for publication.

## Conflict of Interest

The authors declare that the research was conducted in the absence of any commercial or financial relationships that could be construed as a potential conflict of interest.
